# Developing decision support algorithm for hypertension medications for use in a digital therapeutic system

**DOI:** 10.3389/fdsfr.2025.1476998

**Published:** 2025-03-13

**Authors:** Jeannie K. Lee, J. Skye Nicholas, Amani Albadawi, Kathleen C. Insel

**Affiliations:** ^1^ Department of Pharmacy Practice & Science, The University of Arizona R. Ken Coit College of Pharmacy, Tucson, AZ, United States; ^2^ The University of Arizona College of Nursing, Tucson, AZ, United States

**Keywords:** decision support, medication adherence, hypertension, digital therapeutic, older adults, missed dose

## Abstract

**Introduction:**

Adherence to hypertension medications is low among older people, and there is limited guidance on the safety of taking a missed medication. Knowing the window of time when an older individual can safely take a missed medication could improve adherence and reduce the fear of overdosing. We developed and implemented a decision support algorithm for missed hypertension medications in a digital therapeutic system designed to facilitate self-management of hypertension medications and blood pressure among older adults.

**Methods:**

The medication formulary included hypertension medications and doses available in the United States that are safe for use by older people. The decision support algorithm was developed through a literature review of clinical and pharmacokinetic studies and knowledge of the pharmacology of aging (pharmacokinetics and pharmacodynamics). The algorithm was peer-reviewed and checked against available provider and patient instructions about missed hypertension medications. Case scenarios were used to code the algorithm into the app.

**Results:**

A total of 117 hypertension medications and combination products were included in the formulary of the *Medication Education, Decision Support, Reminding, and Monitoring* (MEDSReM^©^) system, a mobile digital therapeutic. The formulary contains medication names and dose options available for selection at onboarding or when medication changes occur. The safe window for taking missed/overdue medication was programmed into the MEDSReM^©^ app for each medication and dosing frequency. The users are presented with “take, snooze, and skip” options when they receive a reminder and open the app to take the medication. If they open the app past the safe window hours, a recommendation to skip that dose and take the next dose at the regular time is presented. If the user decides to take the missed dose, a decision to take or skip the next dose is shown.

**Discussion:**

The MEDSReM^©^ decision support algorithm provides a safe window of time for each hypertension medication for older adults when they need to take their medication after the intended target time has passed. The interdisciplinary collaboration of clinical, technology, and research teams resulted in a novel, older adult-centered, time-specific digital therapeutic intervention to support decision-making about taking missed hypertension medications.

## Introduction

Hypertension is a highly prevalent chronic disease in older people, in which medication is a major factor in lowering blood pressure to optimal levels (Wright et al., 2015; Williamson et al., 2016; Zhang et al., 2021). Multiple medications with proven efficacy for hypertension control are available at low cost in the United States (U.S.), and adherence to these medications can prevent damage to major organs, including the heart, eyes, kidneys, and brain (Whelton et al., 2017; Kleindorfer et al., 2021; Flack et al., 2003; Gottesman et al., 2014; Walker et al., 2019). Yet hypertension medication adherence is a common challenge with multifaceted barriers for patients and with approximately 50% adherence to these effective medications (Viswanathan et al., 2012; Cutler and Everett, 2010; Osterberg and Blaschke, 2005; Vrijens et al., 2008). Thus, our research focuses on improving medication adherence for older people who take hypertension medications using a mobile app as a digital therapeutic system.

Building on the cognitive aging theory-based Multifaceted Prospective Memory Intervention research (Insel et al., 2016; Insel et al., 2013), a technology prototype containing a decision support algorithm for the top 10 most frequently prescribed hypertension medications was tested in a pilot study (Al-Saleh et al., 2024). The prototype was advanced to contain additional features targeting multiple factors related to adherence, including education about hypertension and antihypertensive medications, reminders provided at medication-taking times using age-friendly alarm sounds, and visualization for tracking medication taking and blood pressure. We ensured that strategies incorporated into the system leveraged the older person’s medication taking to be aligned with their established daily routine (Gualtieri et al., 2024). The advanced system called Medication, Education, Decision Support, Reminding, and Monitoring (MEDSReM^©^) also contains an expanded decision support algorithm to include 117 medications and combination products. MEDSReM^©^ is a digital therapeutic system consisting of a smartphone application (app) and a companion website designed to improve self-management of hypertension medications and adherence with improved blood pressure for adults 65 years and older.

The MEDSReM^©^ app contains a hypertension medication formulary and a medication-specific decision support algorithm for older users. The purpose of developing the decision support algorithm with a designated “safe window” to take each hypertension medication was to guide decision-making for older adults when they have missed the usual dosing hour and are trying to determine whether it is still safe to take the missed medication dose given the next scheduled dose. Although taking the medication within an hour of the usual dosing time is ideal, knowing the safe time duration when older adults can take the medication without overdose concerns may improve adherence. We describe the development steps that enabled the MEDSReM^©^ decision support functionality, including the hypertension medication formulary, the algorithm of the safe dosing window for each medication, and coding strategies to communicate decision support to users.

## Materials and methods

The decision support team, led by a clinical pharmacist with geriatric pharmacotherapy and hypertension management expertise, included a nurse investigator with cognitive aging and adherence research expertise, a project coordinator with aging research expertise, and a doctor of pharmacy (PharmD) student interested in aging research. Building the MEDSReM^©^ medication formulary and decision support algorithm for hypertension medications began with the top 10 most frequently prescribed hypertension medications in the U.S. and medications used by field test subjects during the pilot test (Al-Saleh et al., 2024). The full MEDSReM^©^ formulary was subsequently created using all hypertension medications and combination products available in the U.S. that are safe for older adults. Medications potentially inappropriate for older adults included in the 2019 Beers Criteria^®^, such as clonidine and immediate-release nifedipine, were excluded (American Geriatrics Society Beers Criteria, 2019). The formulary, organized by generic drug name, included available brand names and dosages. We used an evidence-based clinical decision-making drug information tool (IBM Micromedex^®^) (Micromedex, 2017) to generate the hypertension medication list. The final formulary list was double-checked using another evidence-based drug information tool (Lexicomp^®^ Lexi-Drugs) (Lexi-drugs, 2024).

The decision support algorithm was created based on a literature review of studies that evaluated missed doses of hypertension medications and the pharmacology of aging, including pharmacokinetic and pharmacodynamic changes that can affect the metabolism and excretion of hypertension medications in older people. We conducted the literature review of missed hypertension medication dose studies using the PubMed search engine to gather information regarding the time to alter the pharmacodynamic properties of hypertension medication after missing a dose and the impact of the missed dose on blood pressure. The pharmacokinetic properties of each medication in the formulary were collected using IBM Micromedex^®^ (Micromedex, 2017) and Lexicomp^®^ (Lexi-drugs, 2024) and included drug onset (time to medication effect upon administration), duration of action (length of time a drug is effective), peak effect (time to peak medication effect), and half-life (time it takes for the concentration of the drug in the body to be reduced by half). The half-life is useful for calculating the estimated time for a medication to reach the steady state (when the concentration of the drug in the body stays consistent) and how long it would take for a medication at a steady state to be eliminated from the body.

The MEDSReM^©^ algorithm containing a safe window of time to take each missed or overdue hypertension medication by older adults was checked a) against commercially published medication information for consumer use, when available; b) against subscription drug information resources for provider use; and c) by peer review. The MEDSReM^©^ safe windows were compared with consumer information to reveal contradictions. Most resources did not contain missed dose information for the public, but we found two sources that somewhat consistently provided this information. The New Zealand Medicines and Medical Devices Safety Authority’s Medsafe (Medicine Information, 2024) provided consumer information on “If you forget to take it,” while WebMD’s Drugs & Medications A–Z (Drugs and Medications, 2024) had a section on “What happens if I miss a dose?” To supplement patient information in the publicly available resources, we searched IBM Micromedex^®^ (Micromedex, 2017) under the “Patient Education: Medication Counseling” section for any available instructions on missed doses of hypertension medications. Within the IBM Micromedex^®^, CareNotes^®^ (CareNotes, 2017) is available for providers to use as handouts for educating patients and care partners. We compared the “Missed dose” section information under “How to Use This Medicine” with our safe window recommendations. In addition, experts in pharmacokinetics, pharmacology, clinical pharmacy, geriatric pharmacy, and geriatric medicine were invited to review and provide feedback and insights, a voluntary request with no incentives. A few safe windows were revised based on these expert opinions and recommendations.

The decision support team worked with the technology partner to code the algorithm and to display understandable app screens for older users. Case examples were created and used for the coding strategy discussions. To further understand existing research on communicating what to do for missed medication doses to patients, we searched the literature for studies on instructing patients. The PubMed and Wiley Online Library databases were used. Search terms included patient education strategy, missed dose education, missed dose of medication, missed dose knowledge, medication adherence communication, patient/physician communication, missed dose communication, patient missed dose knowledge, and missed dose directions.

## Results

The MEDSReM^©^ app contains a hypertension medication formulary and a medication-specific decision support algorithm. The formulary consists of 117 oral hypertension medications and combination products available in the U.S. appropriate for use by older adults. The formulary is organized alphabetically by generic names, with the U.S. brand names, oral formulations, and dose options listed for each generic name. The dose options ranged from 1 to 11, and 41 combination products included two or three medications in one tablet or capsule. Supplementary Appendix A illustrates the MEDSReM^©^ formulary with dose options included.

The decision support algorithm contains designated hours (i.e., a safe window) for older adults to take missed medication doses without overdose concerns. Within the safe window, an option to take or skip a medication dose is available for the user, even if their usual dosing hour has passed. A section of the decision support algorithm containing the safe windows for each medication per frequency of dosing is presented in Table 1. The full decision support algorithm covering all MEDSReM^©^ formulary medications is included in Supplementary Appendix B. The literature search on hypertension medication decision support yielded 30 articles of various types: four safety trials (Kabakci et al., 2010; Nold et al., 1998; Spanos et al., 2014; Tomlinson et al., 2004), six pharmacokinetic studies (Agewall and Kendall, 1997; Bianchetti et al., 1996; Choo et al., 2000; Dimmitt et al., 1998; Fourtillan et al., 1998; Lücker et al., 1990), and 20 clinical trials with intentionally missed doses at the end of the trial (Asmar and Lacourcière, 2000; de Leeuw et al., 2017; Düsing et al., 2013; Elliott et al., 2002; Hermida et al., 2008; Hu et al., 2001; Johnson and Whelton, 1994; Lacourcière and Asmar, 1999; Leenen et al., 1996; Nussinovitch et al., 2002; Ongtengco et al., 2002; Palatini et al., 2010; Redon et al., 2016; Ribeiro et al., 2007; Smilde, 1997; Tan and Leenen, 1999; Vaur et al., 1995; White et al., 2004; Mancia et al., 1999; Myers et al., 2006). None of the studies posed significant changes in blood pressure when the study hypertension medication dose was missed or skipped. This meant a recommendation to skip a missed dose when outside the safe window would not interfere with their blood pressure control.

**TABLE 1:** Example of the MEDSReM^©^ decision support algorithm.

#	Generic name	Brand name (N if none)	Minimum dose (mg)	Maximum dose (mg)	Frequency (per day)	^a^Safe window (hours)
1	Acebutolol	N	200	1,200	1	4
Acebutolol		N	200	600	2	2
2	Aliskiren	Tekturna	150	300	1	6
3	Aliskiren-HCT	Tekturna HCT	150–12.5	300–25	1	4
4	Amiloride	N	5	20	1	4
5	Amiloride-HCT	N	5	50	1	4
6	Amlodipine	Norvasc, Katerzia	2.5	10	1	6
7	Amlodipine–atorvastatin	Caduet	2.5–10	10–80	1	6
8	Amlodipine–benazepril	Lotrel	2.5–10	10–40	1	6
9	Amlodipine–olmesartan	Azor	5–20	10–40	1	6
10	Amlodipine–HCT–olmesartan	Tribenzor	5–12.5–20	10–25–40	1	4
11	Amlodipine–telmisartan	Twynsta	5–40	10–80	1	6
12	Amlodipine–valsartan	Exforge	5–160	10–320	1	6
13	Amlodipine–HCT–valsartan	Exforge HCT	5–12.5–160	10–25–320	1	4
14	Atenolol	Tenormin	25	100	1	4
Atenolol		Tenormin	25	50	2	2
15	Atenolol–Chlorthalidone	Tenoretic 50 and 100	50–25	100–25	1	4
16	Azilsartan	Edarbi	40	80	1	6
17	Azilsartan–chlorthalidone	Edarbyclor	40–12.5	40–25	1	4
18	Benazepril	Lotensin	5	40	1	6
19	Benazepril–HCT	Lotensin HCT	5–6.25	20–25	1	4

^a^ Safe window of hours when older adults can take their missed medications.

HCT, hydrochlorothiazide.

Table 2 presents examples of patient information about missed medication doses available through commercial databases, the New Zealand Safety Authority’s MedSafe (Medicine Information, 2024), and WebMD’s Drugs & Medications A-Z online databases (Drugs and Medications, 2024). The examples of provider and patient instructions on missed doses of hypertension medications from the online subscription resource are also shown in Table 2. Provider-directed information to instruct the patients about missed hypertension medications was vague, inconsistent, and unavailable for 47 unique hypertension medications. For patient instructions, one of the following two recommendations was listed for most hypertension medications: (1) If you miss a dose or forget to take your medicine, take it as soon as you can. If it is almost time for your next dose, wait until then to take the medicine and skip the missed dose. Do not use extra medicine to make up for a missed dose, or (2) Take a dose as soon as you remember. If it is almost time for your next dose, wait until then and take a regular dose. Do not take extra medicine to make up for a missed dose.

**TABLE 2:** Examples of clinician and patient information about missed medication doses.

Rx Name	Consumer medicine information - New Zealand Medicines and Medical Devices Safety Authority	WebMD RxList drugs A-Z consumers - rxlist.com	IBM Micromedex^®^	IBM Micromedex^®^
	Consumer information on “If you forget to take it”	“What happens if I miss a dose?”	Patient education: medication Counseling	CareNotes^®^
Amlodipine	If it is less than 12 h before your next dose, skip the dose you missed and take your next dose when you are meant to. Otherwise, take it as soon as you remember, and then go back to taking your medicine as you would normally.	Take the missed dose as soon as you remember. Skip the missed dose if it is almost time for your next scheduled dose.	Not available	Take a dose as soon as you remember. If it is almost time for your next dose, wait until then, and take a regular dose. Do not take extra medicine to make up for a missed dose.
Hydrochlorothiazide	If it is almost time for your next dose, skip the dose you missed and take your next dose when you are meant to. Otherwise, take it as soon as you remember and then go back to taking your medicine as you would normally.	Take the missed dose as soon as you remember. Skip the missed dose if it is almost time for your next scheduled dose.	Not available	Take a dose as soon as you remember. If it is almost time for your next dose, wait until then, and take a regular dose. Do not take extra medicine to make up for a missed dose.
Lisinopril	If it is almost time for your next dose, skip the dose you missed and take your next dose when you are meant to. Otherwise, take the missed dose as soon as you remember, and then go back to taking your tablets as you would normally.	Take the missed dose as soon as you remember. Skip the missed dose if it is almost time for your next scheduled dose.	Not available	Take a dose as soon as you remember. If it is almost time for your next dose, wait until then, and take a regular dose. Do not take extra medicine to make up for a missed dose.
Losartan	If it is almost time for your next dose, skip the dose you missed and take your next dose when you are meant to. Otherwise, take it as soon as you remember, and then go back to taking your tablet(s) as you would normally.	Take the missed dose as soon as you remember. Skip the missed dose if it is almost time for your next scheduled dose.	Not available	Take a dose as soon as you remember. If it is almost time for your next dose, wait until then, and take a regular dose. Do not take extra medicine to make up for a missed dose.
Metoprolol succinate	If it is less than 12 h before your next dose, skip the dose you missed and take your next dose when you are meant to. Otherwise, take it as soon as you remember, and then go back to taking your medicine as you would normally.	Take the missed dose as soon as you remember. Skip the missed dose if it is almost time for your next scheduled dose.	In the event of a missed dose, counsel the patient to skip the missed dose and maintain a regular dosing schedule.	Take a dose as soon as you remember. If it is almost time for your next dose, wait until then, and take a regular dose. Do not take extra medicine to make up for a missed dose.

The 20 experts (two geriatricians, two pharmacokinetic experts, four PharmDs with collaborative practice in hypertension management, and 12 PharmDs with geriatrics practice) invited to peer-review the formulary and decision support algorithm agreed with the safe windows selection reflecting conservative estimation specific to older adults. Seven articles were found in a literature search to identify the best practices for communicating missed medication dose instructions. Of these, three studies focused on the patient’s understanding and interpretation of medication administration, including instructions on what actions to take when a dose is missed and what patients perceived to be the right course of action when they miss a medication (Davis et al., 2009; Jolles et al., 2013; Lorish et al., 1990). All three studies included people aged 65 years and older, but the mean age of study participants was 40–50 years. Two qualitative studies involved hypertension medications: one study asked patients about their perspectives on hypertension (Jolles et al., 2013), and the other assessed patient understanding of mock medication labels (Davis et al., 2009). The last study targeted rheumatoid arthritis medications (Lorish et al., 1990). The remaining four articles were reviews on strategies implemented by healthcare personnel to educate patients on medication administration that were not specific to hypertension medications (Gilbert et al., 2002; Solem et al., 2003; Newall et al., 2005; Zind et al., 1992) with only two reports targeting missed medication doses (Gilbert et al., 2002; Zind et al., 1992). There was no recent literature and no standardized methods of education to support decision-making by patients on missed medication doses.

**FIGURE 1:**
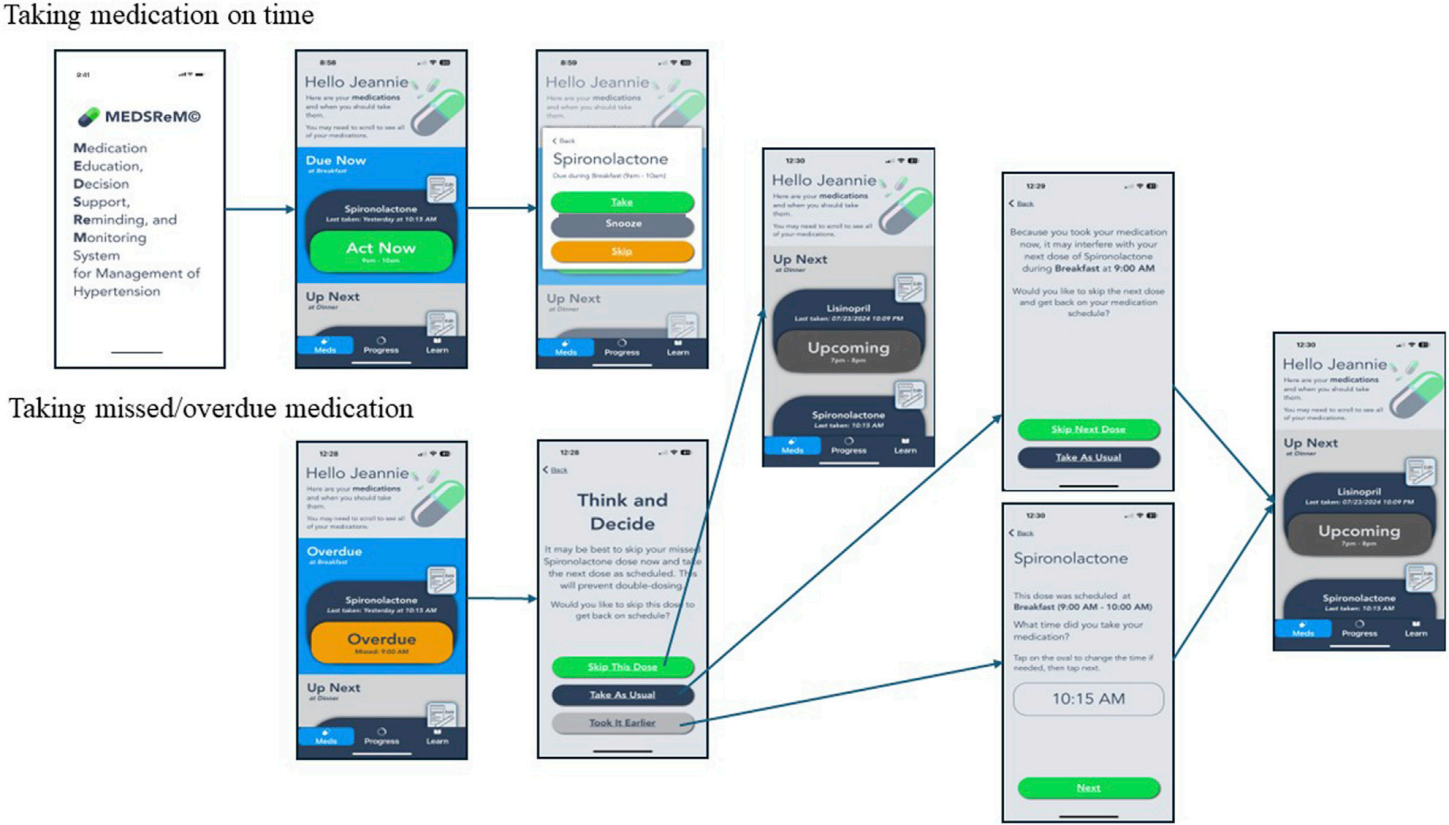
MEDSReM^©^ app medication-taking and decision-support features.

**FIGURE 2:**
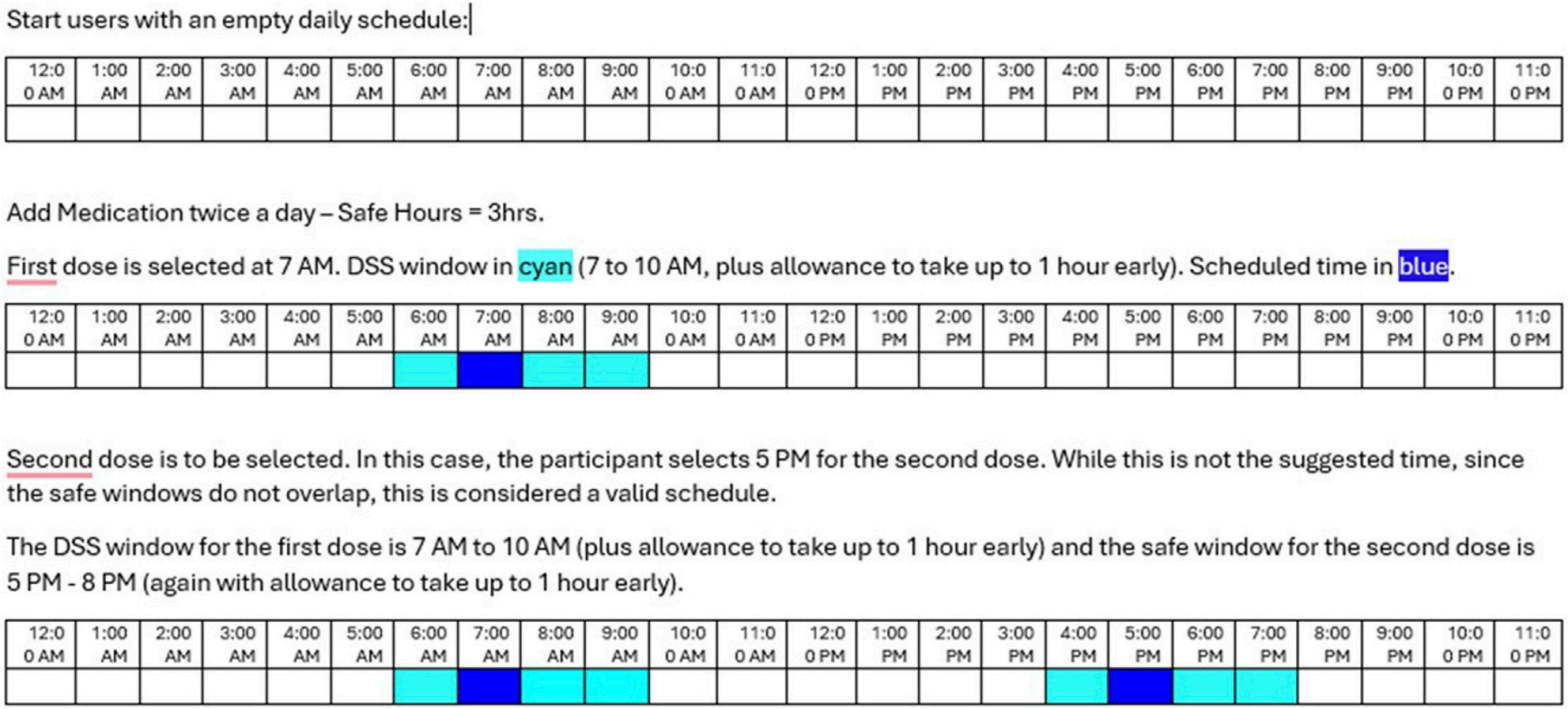
A scenario model of the MEDSReM^©^ decision support scheduling (DSS) for coding.

Figure 1 captures the user app screens for decision support in MEDSReM^©^. The decision support algorithm feature displays information to users when they open the “Meds” tab based on the time of app opening with respect to the dosing window, which is 1 h before the intended dosing time plus the safe window of time in the algorithm for each medication. The options include “Upcoming” when opened too early, “Act Now” when opened on time, and “Overdue” when opened past the dosing window. If the user receives a reminder, opens the app on time within the dosing window, and presses the “Act Now” button, they are presented with “take,” “snooze,” and “skip” options. The “take” option captures medication taking at the moment, while the “snooze” option sends another reminder in 15 min. If the user opens the app past the dosing window and presses the “Overdue” button, they receive a recommendation to skip the medication dose and take the next dose at the regular time. If the user decides to take the overdue medication, the app asks whether they would like to take or skip the next dose to prevent duplicate dosing. The technology partner coded the decision support algorithm into the MEDSReM^©^ app. For this coding, we used a scenario model of a medication taken twice daily that has a 3-h safe window to support the scheduling of the dose (see Figure 2). In addition, the MEDSReM^©^ Decision Support Guide containing three case examples was developed by the investigators and used by our technology partner for the coding process.

## Discussion

An interdisciplinary team created the hypertension medication formulary, decision support algorithm, and coding guide for the MEDSReM^©^ system. To our knowledge, the MEDSReM^©^ decision support algorithm is the first tool developed to help older patients with hypertension make decisions about their missed medication doses, even though there is an expanding market for mobile apps and smart medication adherence products for patient use at home (Park et al., 2019; Faisal et al., 2023). Because clinician information and patient instruction about actions to take for a missed medication dose are inconsistent, not medication-specific, and not age-specific for older adults, we developed the innovative decision support algorithm. This algorithm helps with decision-making about missed hypertension medication doses to improve self-management and potentially improve adherence in older patients.

By design, we made conservative estimates of the safe window hours considering the risks of double-dosing or overdosing in older people, and our peer reviewers agreed with this approach. Even though the MEDSReM^©^ system was developed for those who can self-manage hypertension medications, the conservative estimates addressed the potential risk of frequent changes in health conditions among older adults compared with a younger population. A review and content analysis completed by a research team expressed their concern for the lack of involvement by healthcare providers in medication adherence app development (Ahmed et al., 2018). We found the same results in our initial reviews of existing smartphone apps targeting medication adherence. The MEDSReM^©^ app, including the decision support component, was developed and peer-reviewed through interdisciplinary collaborations among diverse stakeholders involving healthcare experts from pharmacy, nursing, and medicine.

The decision support strategy for actions to be taken when a patient misses a hypertension medication dose was checked against provider and patient education materials. Using the two commercially available online databases with patient information about missed medication doses, Medsafe (Medicine Information, 2024) and Drugs & Medications A–Z (Drugs and Medications, 2024), we reviewed the information for each medication included in the MEDSReM^©^ formulary. Some information was missing from the Medsafe resource, likely due to the differences in available prescription products between the U.S. and New Zealand. We developed a guide and worked closely with our technology partner on coding the decision support algorithm into the MEDSReM^©^ app for seamless delivery and user selection of available options when their medication is overdue. Medication use case scenarios in the guide were formulated to assist with coding and options generated for users.

Overall, there is a lack of up-to-date literature and reliable evidence on educational strategies widely used by healthcare professionals to support decision-making by older patients who missed a medication dose. Similarly, it is unknown which educational strategies for decision-making on missed medication doses are effectively followed by patients. Both instructions for clinicians about advising patients and patient information on managing missed hypertension medication doses were inconsistently available, with most guidance not specific to individual medications. None were age-specific for older adults with hypertension. We found very few review articles that focused on strategies implemented by healthcare personnel to educate patients on medication administration, with only two studies targeting missed medication doses. Various instructions used by healthcare professionals about missed medications ranged from “If you miss one dose or if it is almost time for your next dose, skip the missed dose and continue with your normal schedule” to “Do not double up on doses you have missed” (Gilbert et al., 2002). A study found that although 81% of patients reported adhering to their proton pump inhibitor dosing schedule, the instructions provided by their physicians were frequently not consistent with the product labeling (Solem et al., 2003). This highlights the importance of addressing the prescriber knowledge gap to improve adherence and therapeutic outcomes.

Three studies revealed how some patients manage missed medications, although these studies were also not medication- or age-specific. When assessing patients’ understanding of medication instructions, researchers found that instructions that depicted frequency in hourly intervals (e.g., take one every 12 h) were most often misinterpreted. Time-of-day instructions (e.g., take one in the morning and evening) were better understood by patients compared with the time–frequency (e.g., take one twice a day) or hourly interval instructions (Davis et al., 2009). When asked about the use of blood pressure medications, most participants in a study denied missing a dose but said that they would skip the missed dose due to forgetting or a change in their daily routine and move on to the next scheduled dose; daily routine was considered important for medication adherence (Jolles et al., 2013). Another study found that most patients would skip their missed rheumatoid arthritis medication dose for fear of experiencing side effects from doubling up (Lorish et al., 1990). In the MEDSReM^©^ app, medication-taking time is tied to a daily routine occurring around the same time each day (e.g., breakfast). For missed medication doses (outside the safe window), the decision support feature recommends users skip the missed medication dose and take the next scheduled dose. However, the decision to skip or take the medication is left to the user to allow for self-management and autonomy for our target population with normal age-associated cognitive function.

Limitations to creating a decision support algorithm were having limited information in the literature to draw from and available information being too vague for patient action. Similarly, information for providers to educate patients about missed medication decisions and related patient adherence outcomes was lacking. It was not feasible to test the algorithm for medication effectiveness and safety; however, hypertension medication effectiveness is evidenced by outcomes literature, and conservative estimates of safe windows were used for safety. The decision support algorithm is limited to hypertension medications and combination products, and there is no automated process to generate safe windows for any new hypertension medications or medications used in other conditions. Future research may develop an advanced process via artificial intelligence or machine learning to automate algorithm generation for decision support.

Through interdisciplinary collaboration with people with complementary expertise in pharmacology, cognitive aging, and technology development, we developed and implemented the hypertension medication formulary and decision support algorithm in the MEDSReM^©^ system. It addresses the gap in instructions for missed medication doses by including time-specific and age-specific safe windows for taking the missed medication. Such a tool may be helpful for providers to use when teaching their patients about the safe window of time to take missed medications. Designed with older adult-centered information for older users with hypertension, the MEDSReM^©^ decision support feature may assure older adults to optimize adherence and prevent harm from overdose.

## Data Availability

The original contributions presented in the study are included in the article/Supplementary Material; further inquiries can be directed to the corresponding author.
