# Supplementary material for: Developing decision support algorithm for hypertension medications for use in a digital therapeutic system
Source: Front Drug Saf Regul. 2025 Mar 13;5:1476998. doi: 10.3389/fdsfr.2025.1476998 (PMC12443122; doi:10.3389/fdsfr.2025.1476998)
Supplement: Supplementary file 1 [file DataSheet2.pdf]

Appendix B. MEDSReM Decision Support Algorithm for Hypertension Medications and Combination Products

#	Generic Name	Brand Name (N if none)	Minimum Dose (mg)	Maximum Dose (mg)	Frequency (per day)	*Safe Window (Hours)
1	Acebutolol	N	200	1200	1	4
	Acebutolol	N	200	600	2	2
2	Aliskiren	Tekturna	150	300	1	6
3	Aliskiren-HCT	Tekturna HCT	150-12.5	300-25	1	4
4	Amiloride	N	5	20	1	4
5	Amiloride-HCT	N	5	50	1	4
6	Amlodipine	Norvasc, Katerzia	2.5	10	1	6
7	Amlodipine-Atorvastatin	Caduet	2.5-10	10-80	1	6
8	Amlodipine-Benazepril	Lotrel	2.5-10	10-40	1	6
9	Amlodipine-Olmesartan	Azor	5-20	10-40	1	6
10	Amlodipine-HCT-Olmesartan	Tribenzor	5-12.5-20	10-25-40	1	4
11	Amlodipine-Telmisartan	Twynsta	5-40	10-80	1	6
12	Amlodipine-Valsartan	Exforge	5-160	10-320	1	6
13	Amlodipine-HCT-Valsartan	Exforge HCT	5-12.5-160	10-25-320	1	4
14	Atenolol	Tenormin	25	100	1	4
	Atenolol	Tenormin	25	50	2	2
15	Atenolol-chlorthalidone	Tenoretic 50 and 100	50-25	100-25	1	4
16	Azilsartan	Edarbi	40	80	1	6
17	Azilsartan-Chlorthalidone	Edarbyclor	40-12.5	40-25	1	4
18	Benazepril	Lotensin	5	40	1	6
19	Benazepril-HCT	Lotensin HCT	5-6.25	20-25	1	4
20	Betaxolol	N	5	20	1	6
21	Bisoprolol	N	2.5	20	1	6
22	Bisoprolol-HCT	Ziac	2.5-6.25	20-12.5	1	4
23	Bumetanide	Bumex	0.5	2	2	4
24	Candesartan	Atacand	8	32	1	6
	Candesartan	Atacand	4	16	2	3
25	Candesartan-HCT	Atacand HCT	8-12.5	31-50	1	4
26	Captopril	N	12.5	150	2	4
	Captopril	N	12.5	150	3	2
27	Captopril-HCT	N	25-15	100-50	1	4
28	Carvedilol ER	Coreg CR	10	80	1	6
29	Carvedilol IR	Coreg	3.125	25	2	3
30	Chlorthalidone	N	12.5	25	1	4
31	Diltiazem ER 12-hr Cap	Cardizem	60	120	2	2
32	Diltiazem ER 24-hr Cap	Cardizem CD, Cartia XT, Dilt-XR, Taztia XT, Tiadylt ER, Tiazac	120	360	1	3
33	Diltiazem ER Tab	Cardizem LA, Matzim LA	120	360	1	3
34	Diltiazem IR Tab	Cardizem	30	90	4	1
35	Enalapril	Vasotec	2.5	40	1	6
	Enalapril	Vasotec	2.5	20	2	3
36	Eplerenone	Inspra	50	100	1	6
	Eplerenone	Inspra	50	50	2	3
37	Eprosartan	Teveten	400	800	1	6
	Eprosartan	Teveten	200	400	2	3
38	Eprosartan-HCT	Teveten HCT	600-12.5	600-25	1	4
39	Felodipine ER Tab	N	2.5	10	1	6
40	Fosinopril	N	10	80	1	6
41	Fosinopril-HCT	N	10-12.5	20-12.5	1	4
42	Furosemide	Lasix	10	80	1	4

	Furosemide	Lasix	10	40	2	2
43	Hydralazine	N	10	100	2	3
	Hydralazine	N	10	100	3	2
	Hydralazine	N	10	50	4	1
44	Hydrochlorothiazide (HCT)	N	12.5	50	1	4
	Hydrochlorothiazide (HCT)	N	12.5	25	2	2
45	HCT-Triamterene	Dyazide, Maxzide, Maxzide-25	37.5-25	75/50	1	4
46	Indapamide	N	1.25	5	1	6
47	Irbesartan	Avapro	150	300	1	6
48	Irbesartan-HCT	Avalide	150-12.5	300-25	1	4
49	Isradipine ER	N	5	10	1	6
50	Isradipine IR	N	2.5	5	2	3
51	Labetalol	N	100	800	2	3
	Labetalol	N	100	800	3	2
52	Lisinopril	Zestril, Prinivil	5	80	1	6
	Lisinopril	Zestril, Prinivil	5	40	2	3
53	Lisinopril-HCT	Zestoretic	10-12.5	80-50	1	4
54	Losartan	Cozaar	25	100	1	6
	Losartan	Cozaar	25	50	2	3
55	Losartan-HCT	Hyzaar	100-25	100-25	1	4
56	Metolazone	N	2.5	10	1	4
57	Metoprolol Succinate (XL)	Toprol XL, Kapspargo Sprinkle	12.5	400	1	6
58	Metoprolol Succinate-HCT	Dutoprol	25-12.5	200-25	1	4
59	Metoprolol Tartrate	Lopressor	12.5	200	1	6
	Metoprolol Tartrate	Lopressor	12.5	200	2	3
60	Metoprolol Tartrate-HCT	Lopressor HCT	100-25	200-50	1	4
	Metoprolol Tartrate-HCT	Lopressor HCT	100-25	100-25	2	2
61	Minoxidil	N	5	100	1	4
	Minoxidil	N	5	40	2	2
	Minoxidil	N	5	20	3	1
62	Moexipril	N	7.5	30	1	6
	Moexipril	N	3.75	15	2	3
63	Moexipril-HCT	N	7.5-15	30-50	1	4
64	Nadolol	Corgard	40	320	1	6
65	Nebivolol	Bystolic	5	40	1	6
66	Nicardipine SR	N	30	60	2	3
67	Nicardipine IR	N	20	40	3	2
68	Nifedipine ER	Adalat CC, Afeditab CR, Nifedical XL, Procardia XL	30	90	1	3
69	Nisoldipine Hydrogel ER tablet	Sular	17	34	1	6
70	Nisoldipine Coat-Core ER tablet	Sular	20	60	1	6
71	Olmesartan	Benicar	20	40	1	4
72	Olmesartan-HCT	Benicar HCT	40-12.5	40-25	1	4
73	Perindopril	N	4	16	1	6
	Perindopril	N	2	8	2	3
74	Perindopril-Amlodipine	Prestalia	3.5-2.5	14-10	1	6
75	Pindolol	N	5	30	2	4
76	Propranolol ER	Inderal LA, Inderal XL, InnoPran XL	60	160	1	6
77	Propranolol IR	N	10	80	2	3
	Propranolol IR	N	10	80	3	2
78	Quinapril	Accupril	10	80	1	6
	Quinapril	Accupril	10	40	2	3
79	Ramipril	Altace	2.5	20	1	6

	Ramipril	Altace	1.25	10	2	3
80	Spironolactone	Aldactone	25	100	1	4
	Spironolactone	Aldactone	25	50	2	2
81	Spironolactone-HCT	Aldactazide	25-25	100-100	1	4
	Spironolactone-HCT	Aldactazide	25-25	100-100	2	2
82	Telmisartan	Micardis	20	80	1	6
83	Telmisartan-HCT	Micardis HCT	80-12.5	160-25	1	4
84	Torsemide	N	5	10	1	4
85	Trandolapril	N	2	8	1	6
	Trandolapril	N	1	4	2	3
86	Trandolapril-Verapamil	Tarka	1-180	4-240	1	6
87	Triamterene	Dyrenium	50	200	1	4
	Triamterene	Dyrenium	50	100	2	2
88	Valsartan	Diovan	40	320	1	6
	Valsartan	Diovan	40	160	2	3
89	Sacubitril-Valsartan	Entresto	24-26	97-103	2	3
90	Verapamil ER	Verelan	200	480	1	6
	Verapamil ER	Verelan PM	100	400	1	6
91	Verapamil SR	Calan SR	120	480	1	6
	Verapamil SR	Calan SR	120	240	2	3
92	Verapamil IR	N	40	160	3	2

*Safe window of hours when older adults can take their missed medications

HCT = hydrochlorothiazide, ER = Extended Release, CR = Continuous Release, IR = Immediate Release, hr = hour, Cap = capsule, CD = Controlled Delivery , XT = Extended Release, XR = Extended Release, Tab = tablet, LA = Long Acting, XL = Extended Release, SR = Sustained Release, CC = Calcium Channel blocker, PM = Extended Release
