# Supplementary material for: Developing decision support algorithm for hypertension medications for use in a digital therapeutic system
Source: Front Drug Saf Regul. 2025 Mar 13;5:1476998. doi: 10.3389/fdsfr.2025.1476998 (PMC12443122; doi:10.3389/fdsfr.2025.1476998)
Supplement: Supplementary file 2 [file DataSheet1.pdf]

Appendix A. MEDSReM© Formulary with Dose Options

Generic Name	Brand Name	Dose Option 1 (mg)	Dose Option 2 (mg)	Dose Option 3 (mg)	Dose Option 4 (mg)	Dose Option 5 (mg)	Dose Option 6 (mg)	Dose Option 7 (mg)	Dose Option 8 (mg)	Dose Option 9 (mg)	Dose Option 10 (mg)	Dose Option 11 (mg)
**Acebutolol**		200	400									
**Aliskiren**	Tekturna	150	300									
**Aliskiren-HCT**	Tekturna HCT	150-12.5	150-25	300-12.5	300-25							
**Amiloride**		5										
**Amiloride-HCT**		5-50										
**Amlodipine**	Norvasc, Katerzia	2.5	5	10								
**Amlodipine-Atorvastatin**		2.5-10	2.5-20	2.5-40	5-10	5-20	5-40	5-80	10-10	10-20	10-40	10-80
**Amlodipine-Atorvastatin**	Caduet	2.5-40	5-10	5-20	5-40	5-80	10-10	10-20	10-40	10-80		
**Amlodipine-Benazepril**		2.5-10	5-10	5-20	5-40	10-20	10-40					
**Amlodipine-Benazepril**	Lotrel	5-10	5-20	10-20	10-40							
**Amlodipine-Olmesartan**	Azor	5-20	5-40	10-20	10-40							
**Amlodipine-HCT-Olmesartan**	Tribenzor	5-12.5-20	5-12.5-40	5-25-40	10-12.5-40	10-25-40						
**Amlodipine-Telmisartan**	Twynsta	5-40	5-80	10-40	10-80							
**Amlodipine-Valsartan**	Exforge	5-160	5-320	10-160	10-320							
**Amlodipine-HCT-Valsartan**	Exforge HCT	5-12.5-160	5-25-160	10-12.5-160	10-25-160	10-25-320						
**Atenolol**	Tenormin	25	50	100								
**Atenolol-chlorthalidone**		50-25	100-25									
**Atenolol-chlorthalidone**	Tenoretic 50	50-25										
**Atenolol-chlorthalidone**	Tenoretic 100	100-25										
**Azilsartan**	Edarbi	40	80									

**Azilsartan-Chlorthalidone**	Edarbyclor	40-12.5	40-25									
**Benazepril**		5	10	20	40							
**Benazepril**	Lotensin	10	20	40								
**Benazepril-HCT**		5-6.25	10-12.5	20-12.5	20-25							
**Benazepril-HCT**	Lotensin HCT	10-12.5	20-12.5	20-25								
**Betaxolol**		10	20									
**Bisoprolol**		5	10									
**Bisoprolol-HCT**	Ziac	2.5-6.25	5-6.25	10-6.25								
**Bumetanide**	Bumex	0.5	1	2								
**Candesartan**	Atacand	4	8	16	32							
**Candesartan-HCT**	Atacand HCT	16-12.5	32-12.5	32-25								
**Captopril**		12.5	25	50	100							
**Captopril-HCT**		25-15	25-25	50-15	50-25							
**Carvedilol ER**	Coreg CR	10	20	40	80							
**Carvedilol IR**	Coreg	3.125	6.25	12.5	25							
**Chlorthalidone**		25	50									
**Diltiazem ER 12-hr Cap**		60	90	120								
**Diltiazem ER 12-hr Cap**	Cardizem	30	60	120								
**Diltiazem ER 24-hr Cap**	Tiadylt ER, Tiazac	120	180	240	300	360	420					
**Diltiazem ER 24-hr Cap**	Cardizem CD, Taztia XT	120	180	240	300	360						
**Diltiazem ER 24-hr Cap**	Cartia XT	120	180	240	300							
**Diltiazem ER 24-hr Cap**	Dilt-XR	120	180	240								
**Diltiazem ER Tab**	Cardizem LA	120	180	240	300	360	420					
**Diltiazem ER Tab**	Matzim LA	180	240	300	360	420						
**Diltiazem IR Tab**	Cardizem	30	60	120								
**Diltiazem IR Tab**		30	60	90	120							
**Enalapril**	Vasotec	2.5	5	10	20							
**Eplerenone**	Inspra	25	50									
**Eprosartan**	Teveten	600										

**Eprosartan-HCT**		600-12.5	600-25									
**Felodipine ER Tab**		2.5	5	10								
**Fosinopril**		10	20	40								
**Fosinopril-HCT**		10-12.5	20-12.5									
**Furosemide**	Lasix	20	40	80								
**Hydralazine**		10	25	50	100							
**Hydrochlorothiazide (HCT)**		12.5	25	50								
**HCT-Triamterene**		25-37.5	25-50	50-75								
**HCT-Triamterene**	Dyazide, Maxzide-25	25-37.5										
**HCT-Triamterene**	Maxzide	25-37.5	50-75									
**Indapamide**		1.25	2.5									
**Irbesartan**	Avapro	75	150	300								
**Irbesartan-HCT**		150-12.5	300-12.5									
**Irbesartan-HCT**	Avalide	150-12.5	300-12.5	300-25								
**Isradipine ER**	DynaCirc CR	5										
**Isradipine IR**		2.5	5									
**Labetalol**		100	200	300								
**Lisinopril**	Zestril	2.5	5	10	20	30	40					
**Lisinopril**	Prinivil	5	10	20								
**Lisinopril-HCT**	Zestoretic	10-12.5	20-12.5	20-25								
**Losartan**	Cozaar	25	50	100								
**Losartan-HCT**	Hyzaar	50-12.5	100-12.5	100-25								
**Metolazone**		2.5	5	10								
**Metoprolol Succinate (XL)**	Toprol XL, Kapspargo Sprinkle	25	50	100	200							
**Metoprolol Succinate-HCT**		25-12.5	100-12.5									
**Metoprolol Succinate-HCT**	Dutoprol	25-12.5	50-12.5	100-12.5								
**Metoprolol Tartrate**		25	37.5	50	75	100						
**Metoprolol Tartrate**	Lopressor	50	100									
**Metoprolol Tartrate-HCT**		50-25	100-25	100-50								

**Metoprolol Tartrate-HCT**	Lopressor HCT	50-25										
**Minoxidil**		2.5	10									
**Moexipril**		7.5	15									
**Moexipril-HCT**		7.5-12.5	15-12.5	15-25								
**Nadolol**	Corgard	20	40	80								
**Nebivolol**	Bystolic	2.5	5	10	20							
**Nicardipine SR**		30										
**Nicardipine IR**		20	30									
**Nifedipine ER**	Adalat CC, Procardia XL	30	60	90								
**Nifedipine ER**	Afeditab CR	30	60									
**Nifedipine ER**	Nifedical XL	60										
**Nisoldipine Hydrogel ER Tab**		8.5	17	20	25.5	30	34	40				
**Nisoldipine Hydrogel ER Tab**	Sular	8.5	17	20	25.5	34						
**Olmesartan**	Benicar	5	20	40								
**Olmesartan-HCT**	Benicar HCT	20-12.5	40-12.5	40-25								
**Perindopril**		2	4	8								
**Perindopril-Amlodipine**	Prestalia	3.5-2.5	7-5	14-10								
**Pindolol**		5	10									
**Propranolol ER**	Inderal LA	60	80	120	160							
**Propranolol ER**	Inderal XL, InnoPran XL	80	120									
**Propranolol IR**		10	20	40	60	80						
**Quinapril**	Accupril	5	10	20	40							
**Ramipril**	Altace	1.25	2.5	5	10							
**Spironolactone**	Aldactone, CaroSpir	25	50	100								
**Spironolactone-HCT**		25-25										
**Spironolactone-HCT**	Aldactazide	25-25	50-50									
**Telmisartan**	Micardis	20	40	80								
**Telmisartan-HCT**	Micardis HCT	40-12.5	80-12.5	80-25								
**Torsemide**		5	10	20	100							

**Trandolapril**		1	2	4								
**Trandolapril-Verapamil**	Tarka	1-240	2-180	2-240	4-240							
**Triamterene**	Dyrenium	50	100									
**Valsartan**	Diovan	40	80	160	320							
**Sacubitril-Valsartan**	Entresto	24-26	49-51	97-103								
**Verapamil ER**		100	120	180	200	240	300	360				
**Verapamil ER**	Verelan	120	180	240	360							
**Verapamil ER**	Verelan PM	100	200	300								
**Verapamil SR**	Calan SR	120	180	240								
**Verapamil IR**		40	80	120								

HCT = hydrochlorothiazide, ER = Extended Release, CR = Continuous Release, IR = Immediate Release, hr = hour, Cap = capsule, CD = Controlled Delivery, XT = Extended Release, XR = Extended Release, Tab = tablet, LA = Long Acting, XL = Extended Release, SR = Sustained Release, CC = Calcium Channel blocker, PM = Extended Release
